# Metagenomic analysis of gut microbiota and antibiotic-resistant genes in *Anser erythropus* wintering at Shengjin and Caizi Lakes in China

**DOI:** 10.3389/fmicb.2022.1081468

**Published:** 2023-01-09

**Authors:** Gang Liu, Na Xu, Jiahui Feng

**Affiliations:** School of Life Sciences, Anhui Medical University, Hefei, China

**Keywords:** *Anser erythropus*, antibiotic-resistant genes (ARGs), gut microbiota, metagenomics, Shengjin and Caizi Lakes

## Abstract

Migratory birds are the primary source and reservoir of antibiotic-resistant genes (ARGs) related to their gut microbes. In this study, we performed metagenomics analysis to study the gut microbial communities and ARGs of *Anser erythropus* wintering at Shengjin (SJ) and Caizi (CZ) Lakes. The results showed that bacteria, fungi, viruses, and archaea were the dominant gut microbes. Principal component analysis (PCA) indicated that the microbiota compositions significantly differed between the two populations. Diet may be the most crucial driver of the gut microbial communities for *A. erythropus*. This species fed exclusively on *Poaceae* spp. at Shengjin Lake and primarily on *Carex* spp. at Caizi Lake. Tetracycline, macrolide, fluoroquinolone, phenicol, and peptide antibiotics were the dominant resistant types. ARGs had a significantly higher abundance of operational taxonomic units (OTUs) in the Shengjin Lake samples than in Caizi Lake samples. PCA indicated that most Shengjin Lake samples significantly differed in gut microbiota composition from those obtained at Caizi Lake. This difference in gut microbiota composition between the two lakes' samples is attributed to more extensive aquaculture operations and poultry farms surrounding Shengjin Lake than Caizi Lake. ARGs–microbes associations indicated that 24 bacterial species, commonly used as indicators of antibiotic resistance in surveillance efforts, were abundant in wintering *A. erythropus*. The results revealed the composition and structural characteristics of the gut microbiota and ARGs of *A. erythropus*, pointing to their high sensitivities to diet habits at both lakes. This study also provides primary data for risk prevention and control of potential harmful pathogens that could endanger public health and therefore are of major significance to epidemiological and public health.

## Introduction

Globally, antibiotic pollution is a cause of growing concern and has become a serious threat to public health (Zhao D. D. et al., 2020). With the development of the modern pharmaceutical industry, more and more antibiotics, such as chloramphenicol, quinolones, tetracyclines, vancomycin, and sulfonamides, are being widely used in clinical treatment and in livestock and poultry breeding industries (Mu et al., 2015). As an emergent environmental pollutant, almost all antibiotics are not completely absorbed in nature, and their use causes the selection and spread of antibiotic-resistant genes (ARGs) (Salerno et al., 2022). Thus, ARGs have become a critical concern for public health and have attracted much attention in recent decades (Huang et al., 2017). Agricultural activities, human and animal disease treatments, and the breeding industry are considered potential reservoirs for ARGs (Zhao et al., 2010; Zhu et al., 2013). ARGs can spread or circulate among all ecosystems, such as humans and animals, thus threatening environmental and public health (Huang et al., 2017; Wang et al., 2020; Zhao H. et al., 2020).

Since wild animals are the major source and reservoir of ARGs, which are related to gut microbes, they are used as bioindicators of ARGs in the environment (Li et al., 2016; Cevidanes et al., 2020). Compared to other wild vertebrates, birds, in particular waterbirds, can fly long distances and develop unique diets, lifestyles, and complex physiological traits (Li et al., 2016; Cevidanes et al., 2020). Studies indicated that migratory waterbirds are an important group for the transmission of ARGs in ecosystems, as ARGs may quickly colonize in their gut as a result of the ingestion of polluted food or water (Franklin et al., 2020; Jarma et al., 2021). Migratory waterbirds have a relatively high population density, may encounter ARGs or acquire these genes through contact with each other, and may spread ARGs across large distances (Bauer and Hoye, 2014; Marcelino et al., 2019). Studies indicated that habitats and changes in the avian host diet significantly affect ARGs in the gut of avian species. These avian species, with different ecological patterns, are also likely to play different roles in ARG dispersion (Huang et al., 2017). However, knowledge of the role played by ARGs in the gut of migratory waterbirds is deficient. Only a small number of studies describe how ARGs can change in the same migratory waterbird wintering in different habitats.

As an important wetland indicator species and long-distance migratory waterbird, *A. erythropus* migrate from nesting and breeding areas (Fennoscandian Lapland to northeastern Siberia) in summer to wintering areas (Japan, China, and South Korea). In China, *A. erythropus* mainly stay at the Shengjin (SJ) and Caizi (CZ). Lakes in the Anhui Province in winter. *Carex* and subterranean tubers are their main food in the mudflats of Caizi Lake, whereas *Poaceae* spp. is their main diet in the grasslands of Shengjin Lake (Zhao et al., 2013). The present study hypothesizes that the different food sources that wintering *A. erythropus* consume at the two lakes change the composition and structure of ARGs in their gut. In this study, we performed metagenomics analysis to characterize the gut microbial communities of *A. erythropus* wintering at the two lakes, assessed whether or not their gut ARGs significantly differed between the two lakes, tested if the same migratory waterbird species at the two different wintering habitats played a different role in ARGs, and elucidated the relationship between ARGs and microbes in wintering *A. erythropus*.

## Materials and methods

### Ethical standards

No birds were harmed or killed in this study, and this research was approved by Anhui Medical University (No. 81220270).

### Study areas

Both the Shengjin (30.25°-30.50°N, 116.92°-117.25°E) and Caizi Lakes (30.75°-30.97°N, 117.00°-117.15°E) are located in the southern part of Anhui Province, China, and are shallow lakes connected to the Yangtze River. Both lakes are major international wintering and stopover wetlands for migratory waterbirds in the East Asian–Australasian Flyway (Chen et al., 2011). Both lakes are surrounded by economically developed areas, with the highest population densities in China and with the pursuing of industrial and agricultural activities. However, the two lakes significantly differ from one another in terms of their environmental conditions. Shengjin Lake suffers from severe pollution, overexploitation, in particular due to cage aquaculture operations in recent decades, and pond and low-head dams (Zhao et al., 2013; Li et al., 2015). In recent years, artificial aquaculture enclosures have been demolished at Shengjin Lake; however, many aquaculture and poultry farms still use large quantities of antibiotics for animal treatment. Pollution and overexploitation are less prevalent at Caizi Lake. Mainly paddy fields and a small number of cage aquaculture operations and poultry farms surround Caizi Lake. The water quality (total phosphorus, total nitrogen, permanganate value, chlorophyll-a, and transparency) of Caizi Lake is better than that of Shengjin Lake (Gu et al., 2014).

### Sample collection and avian species determination

Twenty fecal samples were collected from paddy fields at the two lakes (10 samples each) in December 2021. A large sample size of more than 100–150 geese was selected to collect fecal samples at foraging sites from the two lakes. Foraging behavior was observed using telescopes or binoculars before the fecal samples were collected. After the geese finished foraging and had defecated, we immediately collected the fresh fecal samples. To avoid individual repetition, human disturbance, and soil contamination, the distance between successive fecal samples was at least 5 m, and we collected only the center of each fecal mass. All samples were immediately transported to the laboratory and stored at −80°C (Dong et al., 2019; Xiang et al., 2019; Liu et al., 2020).

A DNA Stool Mini kit was used to extract the DNA from the avian fecal sample, and the *cox1* gene of the mitochondrial genome was used to confirm the avian species. The primers (BIRDF1: 5′-TTC TCC AAC CAC AAA GAC ATT GGC AC-3′ and BIRDR1: 5′-ACG TGG GAG ATA ATT CCA AAT CCT G-3′) were used to identify the goose species *via* polymerase chain reaction (PCR) amplification (Liu et al., 2020). The successive cycling conditions of PCR amplification consisted of denaturation at 95°C for 5 min, 35 cycles of denaturation at 95°C for 30 s, annealing at 55°C for 45 s, extension at 72°C for 1.5 min, and a final extension at 72°C for 10 min. The PCR products were sequenced, and all resulting sequences were blasted in GenBank to ensure that all fecal samples in this study were taken from *A. erythropus* alone.

### Illumina high-throughput sequencing and bioinformatics analysis

Metagenomic sequencing and analysis of the fecal samples were conducted using an Illumina HiSeq 2500 at OE Biotech Co., Ltd. (Shanghai, China). MEGAHIT (v1.1.2) software was used to perform metagenomic assembly after the recording of valid reads. Clean reads were aligned against the non-redundant gene set (95% identity) using Bowtie 2 (v2.2.9). The gene set representative sequence of the fecal samples in this study was annotated with the Kyoto Encyclopedia of Genes and Genomes (KEGG), Swiss-Prot, Non-Redundant Protein Sequence Database (NRDB), Clusters of Orthologous Groups of proteins (COGs), and Gene Ontology (GO) databases. KEGG, Carbohydrate-Active Enzymes (CAZy), Evolutionary Genealogy of Genes: Non-supervised Orthologous Groups (eggNOG) Database, and Comprehensive Antibiotic Resistance Database (CARD) of the unigenes were obtained. The linear discriminant analysis effect size (LEfSe) method was used to compare the two groups in order to determine the differentially abundant taxonomic features by using the non-parametric Kruskal–Wallis rank sum test.

### Statistical analysis

Differences in the gut microbial communities and ARGs between the two geese populations were analyzed using the non-parametric Kruskal–Wallis rank sum test. All statistical analyses and associated plots, such as principal component analysis (PCA), pairwise Spearman's correlations, R scores, and *p*-values, were performed using GraphPad Prism v 7.0, SPSS 22 and Oebiotech tools available at https://cloud.oebiotech.cn/task/.

## Results

### Composition of microbial communities

**Figure 1:**
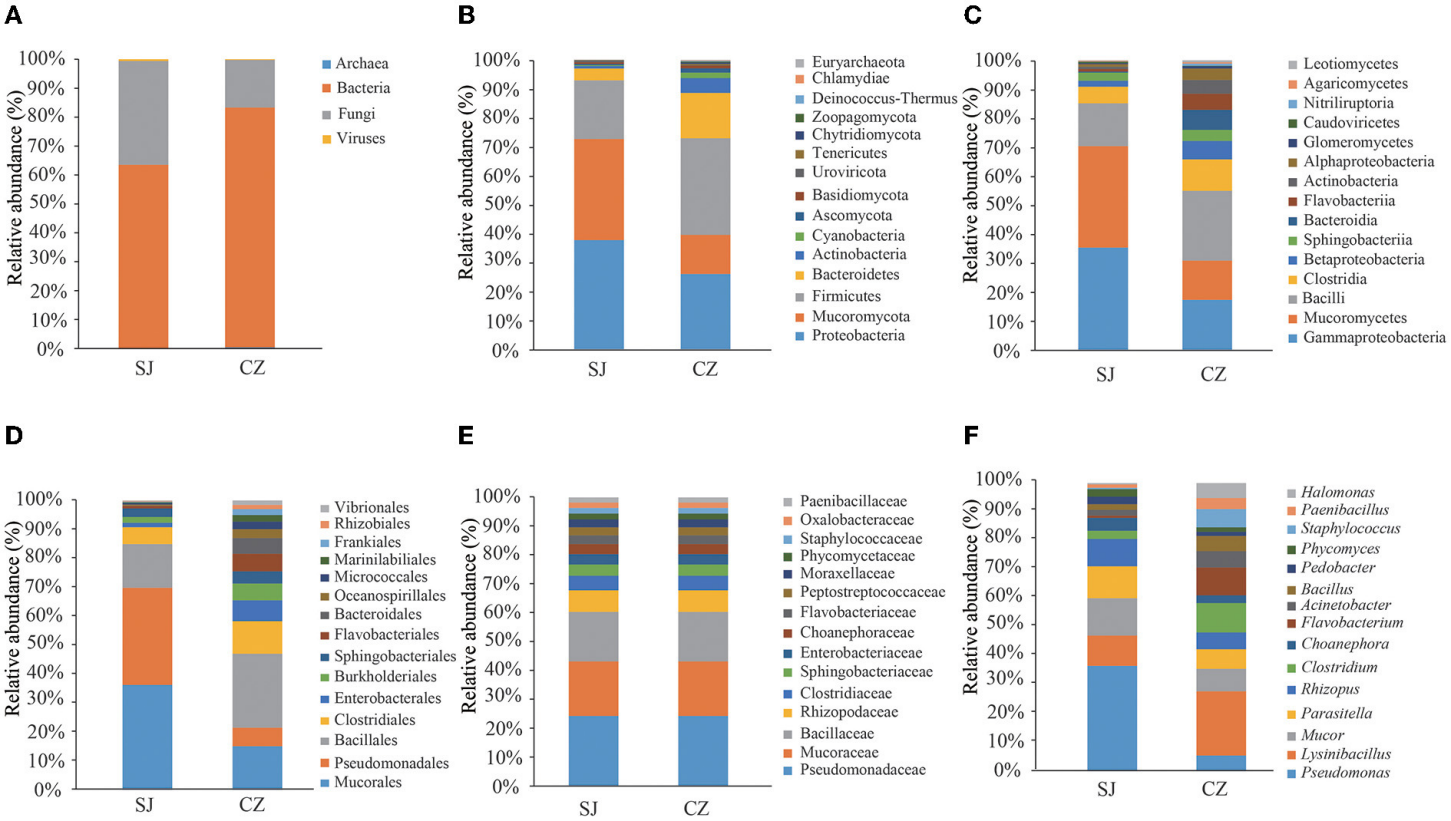
Taxonomic analyses at the levels of Kingdom **(A)**, Phyla **(B)**, Classes **(C)**, Orders **(D)**, Families **(E)**, and Genera **(F)** of *Anser erythropus* wintering at Shengjin (SJ) and Caizi (CZ) Lakes.

The Illumina MiSeq 2500 sequencing run produced 1,186,628,964 raw reads. After the removal of low-quality reads, 1,182,900,206 clean reads corresponding to 7,935,970 operational taxonomic units (OTUs) were retained. Of the OTUs, 39.48% were found in both populations. Geese from Shengjin Lake had 10.02% unique OTUs, while geese from Caizi Lake had 11.02% unique OTUs. A Venn analysis indicated that 2,787 unique OTUs were identified in the 20 samples (Supplementary Figure 1). Four kingdoms, 164 phyla, 299 classes, 537 orders, 1,096 families, and 3,558 genera were identified in the gut microbes of *A. erythropus*. Bacteria, fungi, viruses, and archaea were the dominant gut microbes, accounting for 71.35, 28.06, 0.38, and 0.21% of the OTUs, respectively (Figure 1A). The abundance of bacteria was higher than those of archaea, eukaryotes, and viruses. Proteobacteria (32.69%), Mucoromycota (26.06%), Firmicutes (25.34%), Bacteroidetes (8.62%), and Actinobacteria (2.59%) were the dominant microbial phyla (Figure 1B). Gammaproteobacteria, Mucoromycetes, and Bacilli were the dominant microbial classes, accounting for 26.93, 2.545, and 1.75%, respectively (Figure 1C). Mucorales (1.273%) and Pseudomonadales (1.05%) were the dominant microbial orders (Figure 1D). Pseudomonadaceae, Mucoraceae, Bacillaceae, Rhizopodaceae, and Clostridiaceae were the dominant microbial families, accounting for 20.36, 15.73%, 14.32, 6.22, and 4.20%, respectively (Figure 1E). *Pseudomonas* (18.95%), *Lysinibacillus* (9.50%), *Mucor* (7.87%), and *Parasitella* (6.76%) were the dominant microbial genera (Figure 1F).

### Diversity and differences in taxa of microbes

**Figure 2:**
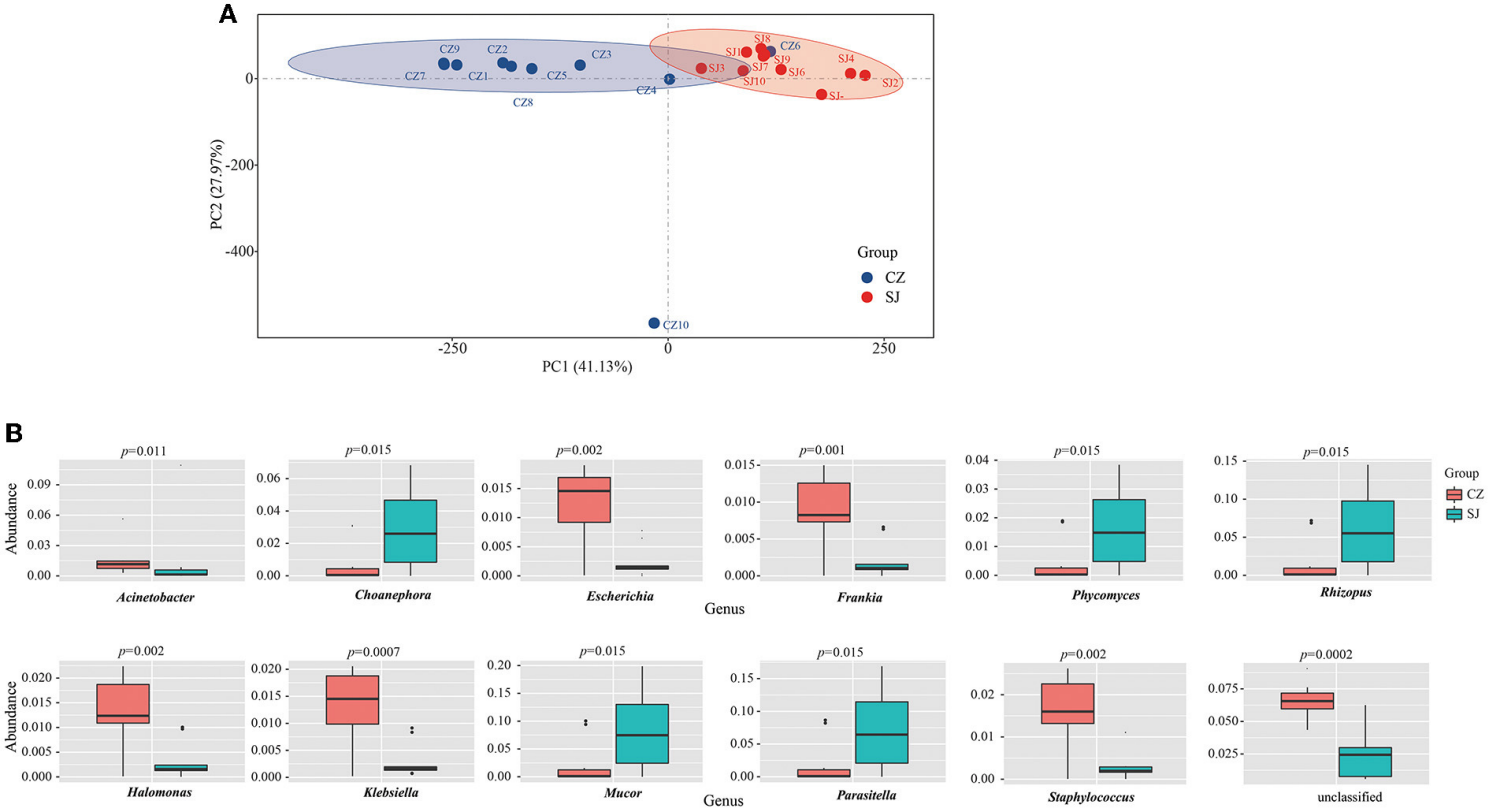
Principal component analysis (PCA) **(A)** and different genera **(B)** of the gut microbes of *Anser erythropus* sampled from Shengjin (SJ) and Caizi (CZ) Lakes.

The results of PCA indicated that the microbiota compositions significantly differed between the two populations, and all the individuals were well-matched with their lakes (Figure 2A). The top 12 genera of microbial abundance were found in all the samples between the two lakes (Figure 2B). *Acinetobacter, Escherichia, Frankia, Halomonas, Klebsiella*, and *Staphylococcus* had a significantly higher abundance of OTUs in the Shengjin Lake samples than in the Caizi Lake samples, while *Choanephora, Phycomyces, Rhizopus, Mucor*, and *Parasitella* were more abundant in the Caizi Lake samples than in the Shengjin Lake samples.

### Functions of the gut microbes

**Figure 3:**
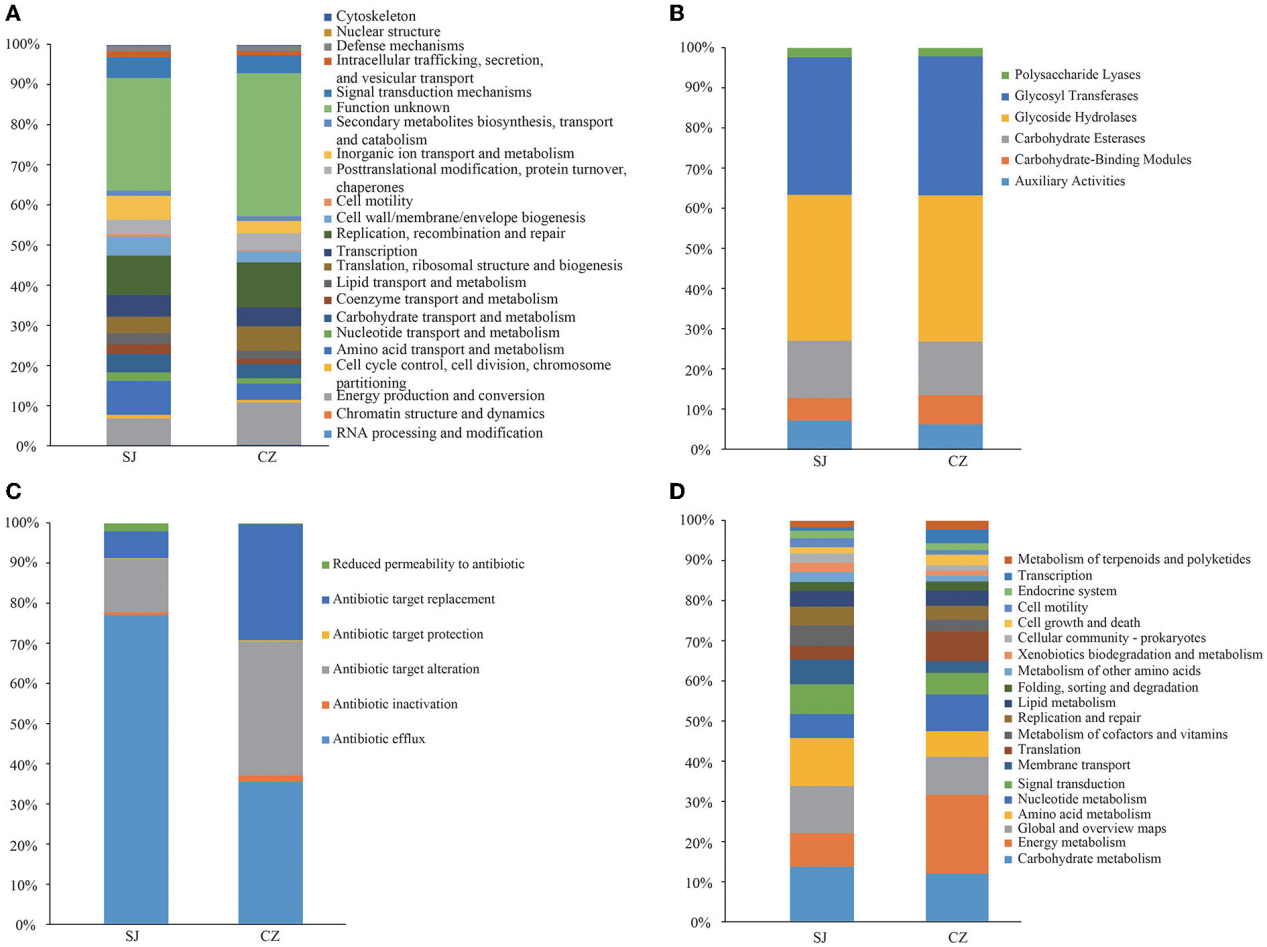
Functional predictions of *Anser erythropus* gut microbial communities from Shengjin (SJ) and Caizi (CZ). Lakes based on the Evolutionary Genealogy of Genes: Non-supervised Orthologous Groups (eggNOG)_description **(A)**, Carbohydrate-Active Enzymes (CAZy) **(B)**, Comprehensive Antibiotic Resistance Database (CARD) **(C)**, and Kyoto Encyclopedia of Genes and Genomes (KEGG) **(D)** analyses.

The gut microbial functional pathways were annotated based on the eggNOG, CAZy, CARD, and KEGG databases. Most of the gene functions remained unknown (30.74%) according to the eggNOG annotation results. The eggNOG annotation results showed that replication, recombination, and repair (10.32%); energy production and conversion (7.86%); and amino acid transport and metabolism (6.99%) were the main functions (Figure 3A). The CAZy annotation results showed that the glycoside hydrolases (36.33%), glycosyl transferases (34.38%), and carbohydrate esterases (13.97%) were the primary functions (Figure 3B). Based on the CARD annotation results, antibiotic efflux was the most abundant function (72.21%) (Figure 3C). The KEGG results showed that carbohydrate metabolism (11.96%), energy metabolism (11.05%), and global and overview maps (10.16%) were most abundant in the KEGG functions (Figure 3D). Between the gut microbial communities at the two lakes, there were no differences in eggNOG_description (*p* = 0.06), CAZy (*p* = 0.38), and CARD (*p* = 0.24). However, the KEGG results indicated a significant difference between the two communities (*p* = 0.02).

### Abundance, diversity, network analysis, and differences in taxa of ARGs

**Figure 4:**
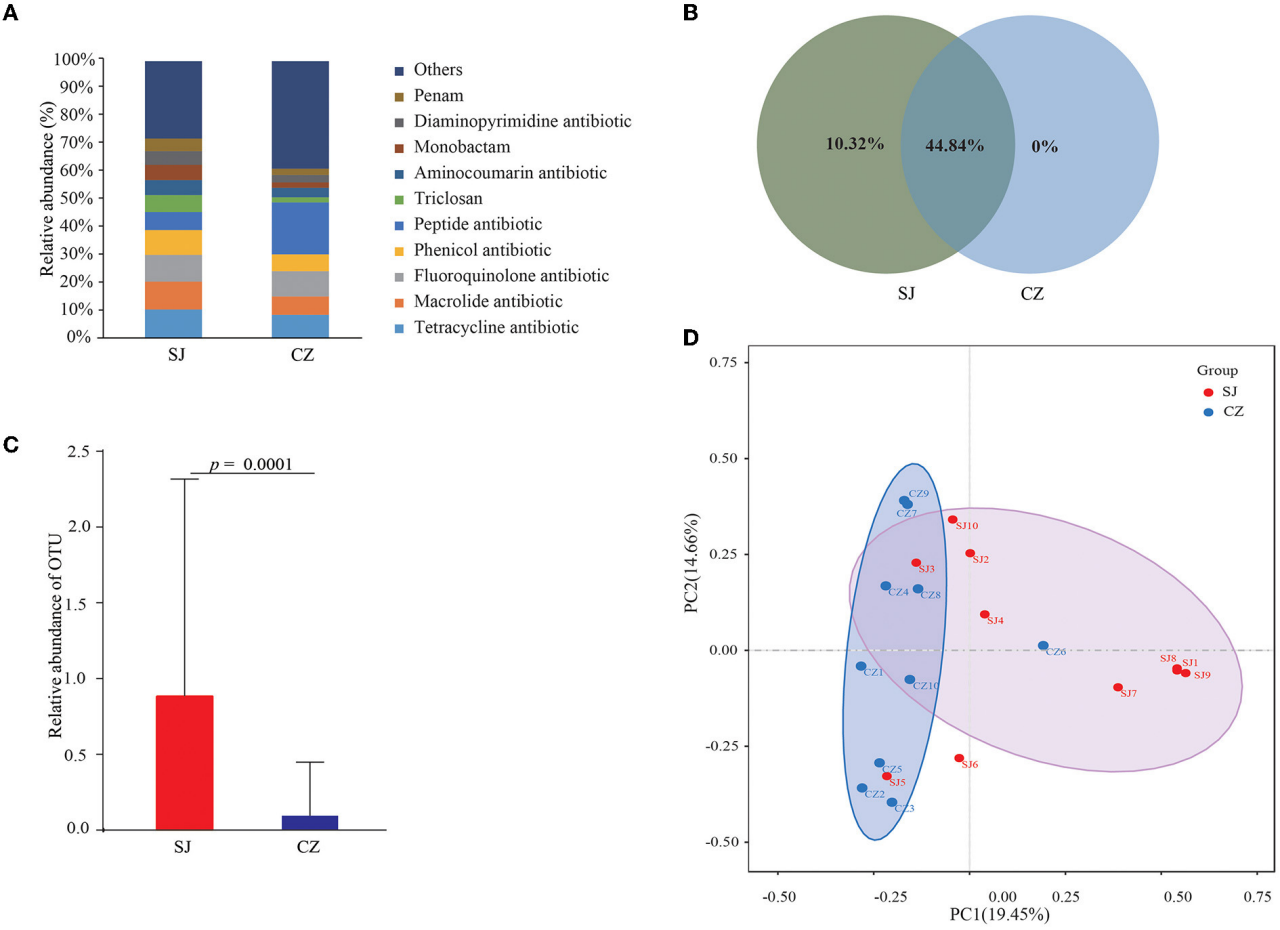
Fractions of relative abundance of genes **(A)**, Venn analysis **(B)**, difference in the relative abundance of operational taxonomic units (OTUs) **(C)**, and principal component analysis (PCA) **(D)** of *Anser erythropus* sampled from Shengjin (SJ) and Caizi (CZ) Lakes.

**Figure 5:**
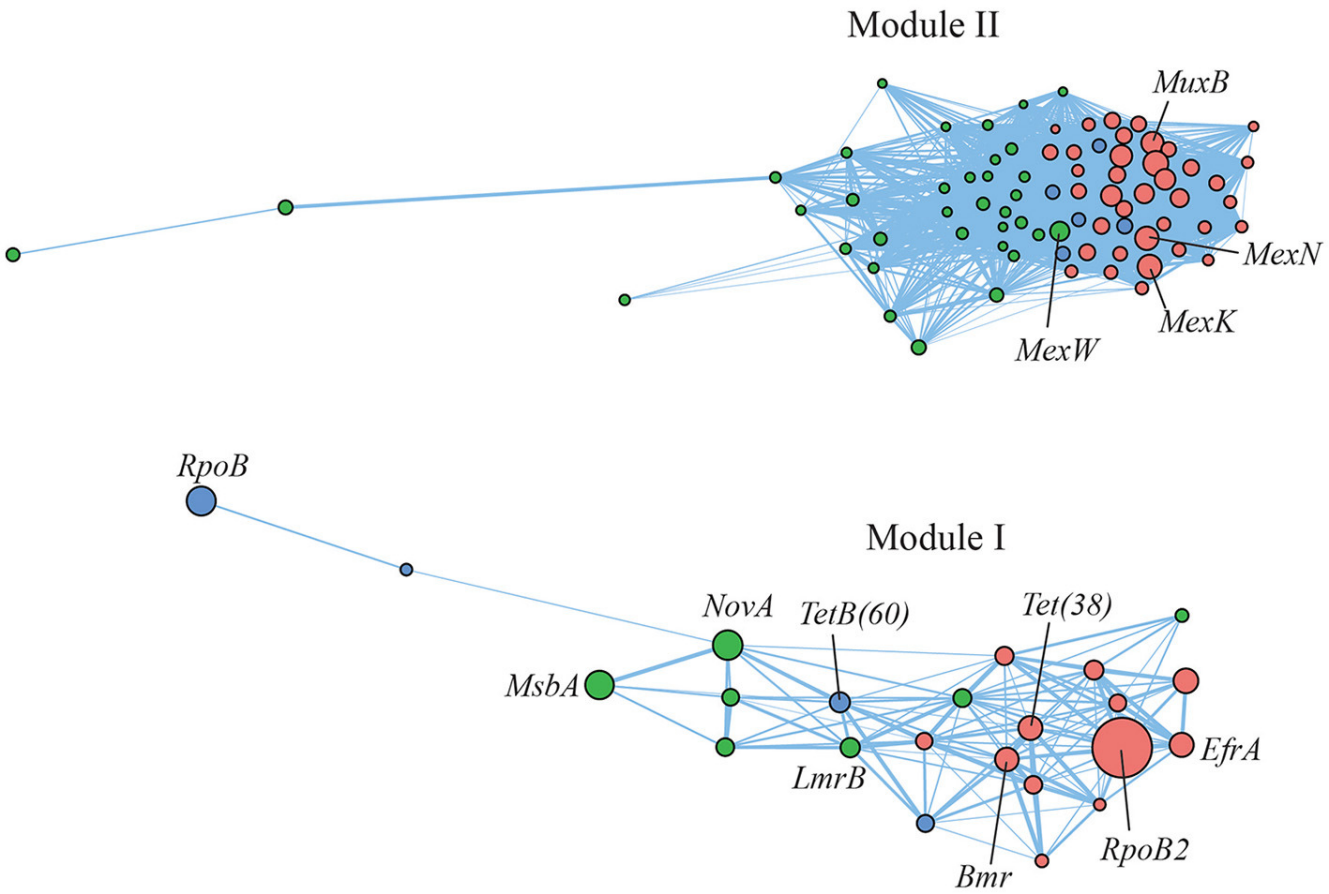
Correlation network analysis of the gut antibiotic-resistant genes (ARGs) of *Anser erythropus* wintering at Shengjin (SJ) and Caizi (CZ) Lakes.

**Figure 6:**
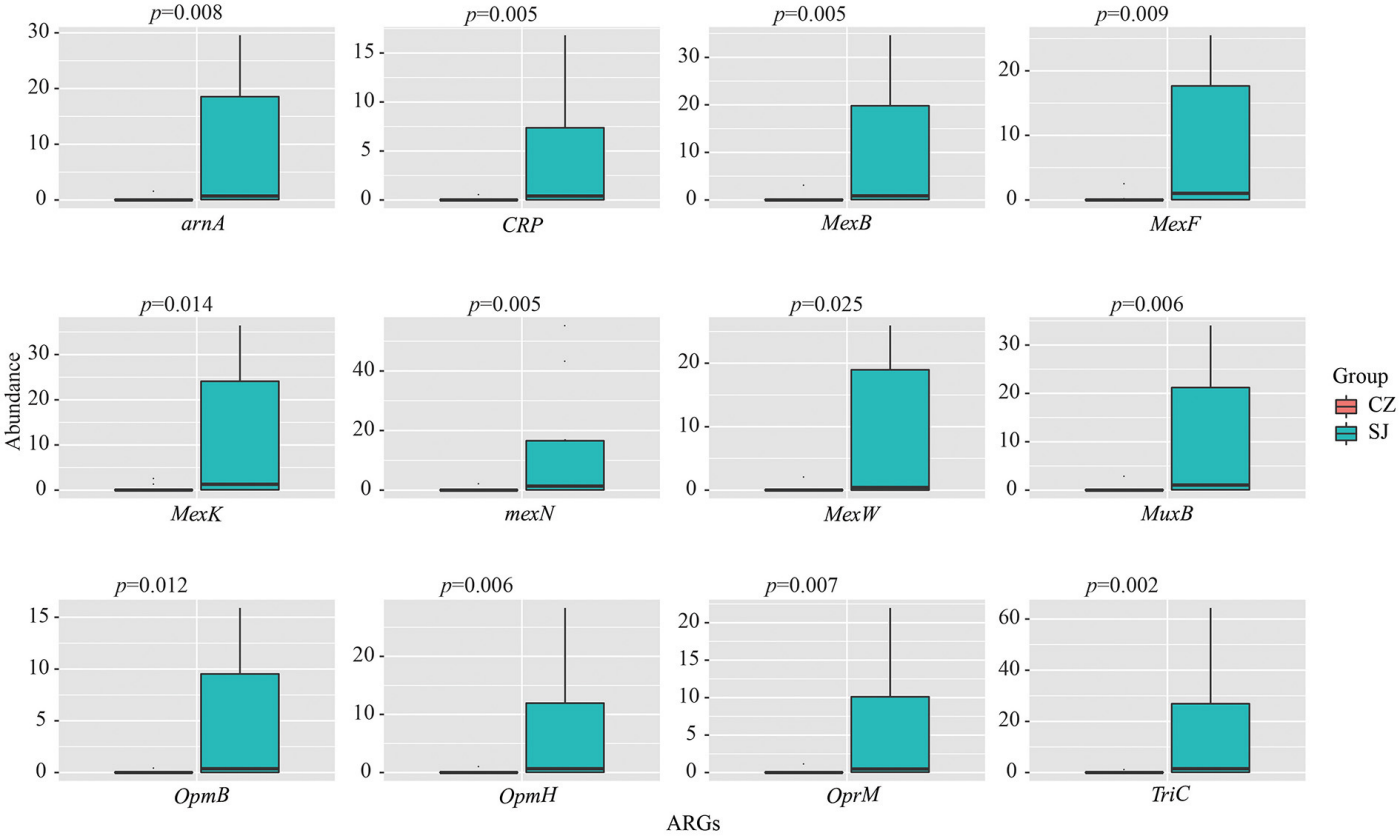
The top 12 antibiotic-resistant genes (ARGs) in *Anser erythropus* sampled from Shengjin (SJ) and Caizi (CZ) Lakes.

In total, 2,218 ARGs were detected in the gut of *A. erythropus*, categorized into 34 resistant types. Tetracycline (14.55%), macrolide (13.72%), fluoroquinolone (13.41%), phenicol (12.34%), and peptide (10.14%) antibiotics were the dominant resistant types (Figure 4A). Of all the ARGs, 44.84% were found in both populations. Geese from Shengjin Lake had 10.32% unique ARGs, while those from Caizi Lake had none (Figure 4B). The ARG results indicated that the relative abundance of OTUs significantly differed between the samples from the two lakes (*p* = 0.0001) (Figure 4C). All samples were further analyzed using the PCA method according to the ARG abundance. All the Shengjin Lake samples, except for SJ5, were grouped together and significantly differed from the Caizi Lake samples, except for CZ6 (Figure 4D). The results of the top 100 ARGs' network analysis can be split into two major modules that consisted of 23 and 77 total nodes in each (Figure 5). *RpoB2* was the hub of Module I, whereas *MexK, MexN, MuxB*, and *MexW* were the hubs of Module II. The top 12 ARGs were found in all the samples from the two lakes and had a significantly higher abundance of OTUs in the Shengjin Lake samples than in the Caizi Lake samples (Figure 6).

### ARGs–microbe association analysis

**Figure 7:**
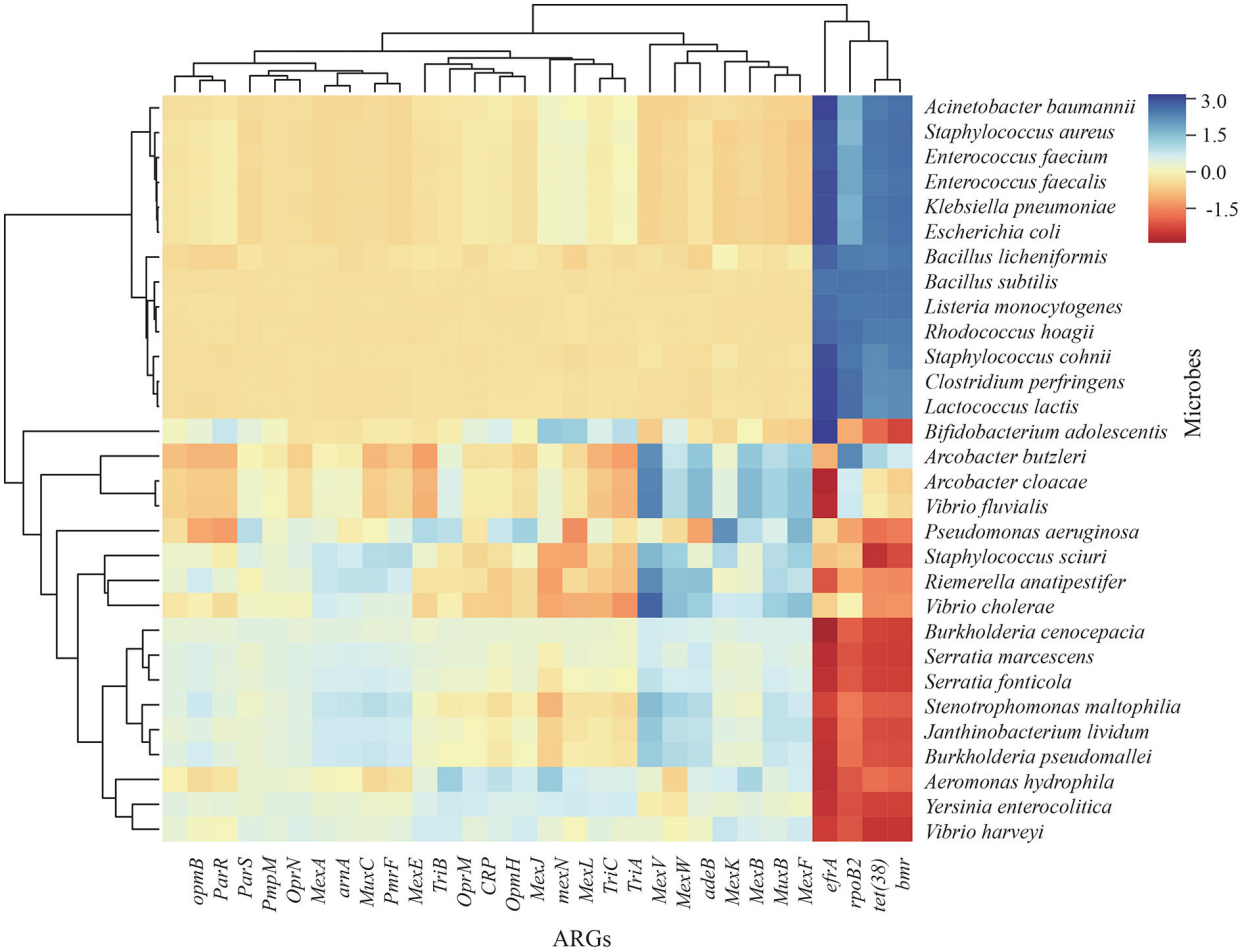
Correlation heatmap of microbes and antibiotic-resistant genes (ARGs) in the gut of *Anser erythropus* sampled from Shengjin (SJ) and Caizi (CZ) Lakes.

Correlation analysis confirmed associations between ARGs and bacteria. One hundred and four bacterial species represented most of the ARGs. Twenty-four bacterial species, including *Arcobacter cloacae, Vibrio harveyi, Vibrio cholerae, Stenotrophomonas maltophilia, Serratia marcescens, Serratia fonticola*, and *Riemerella anatipestifer*, commonly used as bioindicators of antibiotic resistance in surveillance efforts, were abundant in wintering *A. erythropus*. The frequencies of *Efra, Rpob2, Tet(38)*, and *Bmr* were most correlated with the abundance of microbes (Figure 7).

## Discussion

In our study, Proteobacteria, Mucoromycota, and Firmicutes were the most abundant gut microbiota in *A. erythropus*. During the wintering period, geese require a great amount of energy to cope with the cold, and Proteobacteria may play an important role in energy accumulation (Dong et al., 2019). Mucoromycota are common fungi in the highly versatile metabolic gut system, and these fungi may act as powerful microbial cell factories for biorefinery applications (Dzurendova et al., 2022). Firmicutes could help the geese to absorb nutrients and intake energy from the metabolism of polysaccharides, fatty acids, carbohydrates, and sugars (Flint et al., 2008; Tap et al., 2009). The gut microbial composition and characteristics of birds are different from those of other vertebrates due to complex and unique avian physiological traits, diets, and migration strategies (Song et al., 2020; Li et al., 2021). Most studies have highlighted that the avian gut microbiome is influenced by diet, age, genetics, captivity, and changes in their habitats (Hammons et al., 2010; Waite and Taylor, 2014; Barbosa et al., 2016; Wang et al., 2016; Díaz-Sánchez et al., 2019). The diet was the primary driver of the gut microbial community structure for the host, as the food source of wintering *A. erythropus* was different at the two lakes. The species fed exclusively on *Poaceae* spp. at Shengjin Lake and primarily on *Carex* spp. at Caizi Lake (Wang et al., 2012; Zhao et al., 2012). This feeding behavior suggested that the different food intakes of wintering *A. erythropus* at the two lakes changed their gut microbial community compositions. It was reported that migratory birds show a strong-site fidelity for wintering and breeding areas and often return to the same site each year in winter (Møller and Szép, 2011). This phenomenon may be related to the fact that *A. erythropus* wintering at Shengjin Lake migrated from a different breeding location, compared to those at Caizi Lake (Liu et al., 2020). However, no detailed information regarding the migration routes of *A. erythropus* wintering at the two lakes is available. The gut microbial communities and functions of wintering *A. erythropus* differed between the two lakes, as supported by the 16S rRNA gene sequencing results (Liu et al., 2020). However, the dominant microbial phyla were different from the 16S rRNA gene sequencing results, in that Proteobacteria was the most abundant phyla in the metagenomic results, while Firmicutes was the highest in the 16S rRNA gene sequencing results (Liu et al., 2020).

Antibiotic-resistant genes have been considered an emerging environmental contaminant in aquaculture, wastewater, soil, sewage plants, and livestock feces, threatening worldwide public health (Durso et al., 2012; Cevidanes et al., 2020). Previous studies showed that ARGs have increased in the environment due to animal and human disease treatments and agricultural activities. The spread and aggregation of ARGs into resistant pathogens have challenged life-saving antibiotic therapies in recent decades (Zhu et al., 2013; Cevidanes et al., 2020; Wang et al., 2020). ARGs can be transferred among species in the wild through several mobile genetic elements, such as integrons, plasmids, and transposons (Bellanger et al., 2014; Blair et al., 2015; Chen et al., 2015; Mu et al., 2015; Kipkorir et al., 2020). Wild birds are under the selective pressure of antibiotics, in that not only do these antibiotics kill beneficial gut bacteria, but they also alter the composition, structure, and function of the gut microbiome, even causing some bacteria to completely disappear from the gut (Wang et al., 2017). Migratory birds can acquire and transmit ARGs from contaminated environments along migration flyways (Foti et al., 2011). ARGs in the gut of migratory birds are considered an emerging public health problem since their gut microbes can carry and spread ARGs to humans, and these ARGs render antibiotic drugs obsolete (Yuan et al., 2021). As a long-distance migratory waterbird, *A. erythropus* is found in various ecosystems; however, ARGs in this species have received little attention. This study is the first to explore ARGs in *A. erythropus* wintering at the two Lakes. The abundance of ARGs in wintering *A. erythropus* was found to be higher at Shengjin Lake than at Caizi Lake. This result was consistent with the abundance of their gut microbial communities and may be due to the heterogeneous environmental factors of the two lakes (Liu et al., 2020). It was reported that microbes can harbor ARGs, selected by a variety of antibiotics, acquire antibiotic resistance, frequently switch hosts within the same microbial community or among different microbial communities, and spread from the environment to migratory waterbirds (Sommer et al., 2009; Bonnedahl and Jarhult, 2014; Liu et al., 2016). ARGs are not only resistant to antibiotics but are also critical to the environment. A recent study demonstrated that the external living environment affects the gut ARG diversities and the origin of ARGs in the guts of waterbirds in the environment (Jarma et al., 2021). With the rapid rates of economic, population, and consumption growth, ARGs have been increasingly used for agriculture and aquaculture around Shengjin and Caizi Lakes (Li et al., 2015). Therefore, the ARGs in the gut of *A. erythropus* appear to originate from the habitats at the two lakes. A higher ARG diversity was observed in the geese wintering at Shengjin Lake than at Caizi Lake, probably because aquaculture operations and poultry farms affected Shengjin Lake more than Caizi Lake. The CRP and cpxR types were highly abundant in the gut of *A. erythropus*. The CRP type is a regulator that modulates bacterial antibiotic resistance, and its mutants can increase the resistance ability of bacterial strains (Nishino et al., 2008). CpxR is identified as a regulator of the cell envelope stress response and acts as an activator to enhance the expression of the efflux pump and improve drug resistance (Tian et al., 2016).

The gut microbiota and ARGs in *A. erythropus* wintering at the two lakes were correlated with the physicochemical properties of the two lakes. It was previously reported that the concentrations of chemical ions in the surface and groundwater of the study area were higher than those of atmospheric precipitation, while total phosphorus, total nitrogen, chlorophyll-a, and transparency values of the surface water pointed to eutrophication (Cui et al., 2021; Gao et al., 2022; Zhao et al., 2022). The trophic state index and bacterial eutrophic index values showed that the two lakes were slightly eutrophic (Gao et al., 2022; Zhao et al., 2022). These previously reported results indicate that the physicochemical properties of these two lakes affect the structure and function of the gut microbiota and ARGs in *A. erythropus*. These inferences remain to be validated in the future.

Wild animals serve as important carriers of ARGs and play a vital role in their spread worldwide. Migratory birds serve as an important reservoir of resistant bacteria and ARGs and can carry them over long distances, even across continents. Thus, migratory birds may operate as a transmission route and are partly responsible for the global dissemination of ARGs. This study is the first step in describing the characterization of the presence of ARGs in the gut of *A. erythropus* wintering at the two lakes. The presence of elevated levels of ARGs in *A. erythropus* feces poses a significant risk of acquisition by human pathogens. *A. erythropus* has an enormous potential to spread ARGs to places where humans are most vulnerable to them. These results underscore the need to limit human exposure to bird feces and to proactively manage the associated risks. Although the origin of ARGs in *A. erythropus* is still to be clarified, as wild birds are not directly exposed to antibiotics, contact with sewage, soil, or animal manure may affect humans. According to this study, these geese carry ARGs during their migration and become a major source of ARGs in the local environment. Overall, this study suggests that different food resources and habitat heterogeneity affect the microbiota community's composition and structure in *A. erythropus* and their exposition and acquisition of ARGs. However, there are some limitations in this study that need to be addressed in future studies. First, its relatively small sample size should be increased so as to represent a more spatiotemporal sample. Second, soil, water, plant, and air samples are required to correlate *A. erythropus* and their gut microbial communities as well as ARG compositions with environmental factors.

## Data availability statement

The datasets presented in this study can be found in online repositories. The names of the repository/repositories and accession number(s) can be found below: https://www.ncbi.nlm.nih.gov/genbank/, PRJNA894380.

## Ethics statement

The animal study was reviewed and approved by this research was approved by Anhui Medical University (No. 81220270).

## Author contributions

GL designed the experiments, analyzed the data, and wrote the manuscript. JF and NX performed the experiments. All authors contributed to the article and approved the submitted version.
