# Supplementary material for: Metagenomic analysis of gut microbiota and antibiotic-resistant genes in Anser erythropus wintering at Shengjin and Caizi Lakes in China
Source: Front Microbiol. 2023 Jan 9;13:1081468. doi: 10.3389/fmicb.2022.1081468 (PMC9868308; doi:10.3389/fmicb.2022.1081468)
Supplement: Supplementary file 1 [file Image_1.pdf]

Supplementary information

Supplementary Figure

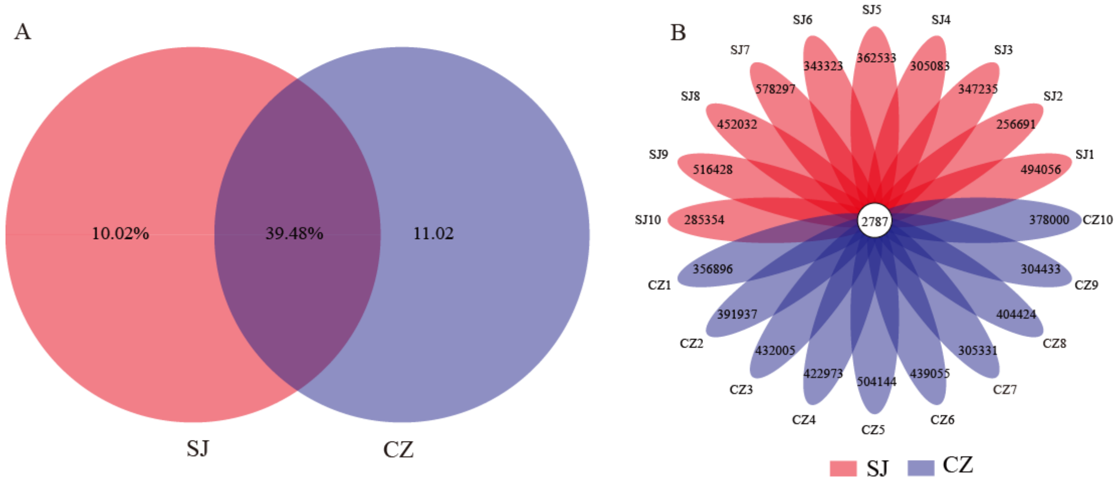

Supplementary Figure 1 Venn analysis (A) unique core OTUs (B) of gut microbiome from *A. erythropus* wintering at Shengjin (SJ) and Caizi (CZ) Lakes.
